# Optimized and Automated Radiosynthesis of [^18^F]DHMT for Translational Imaging of Reactive Oxygen Species with Positron Emission Tomography

**DOI:** 10.3390/molecules21121696

**Published:** 2016-12-09

**Authors:** Wenjie Zhang, Zhengxin Cai, Lin Li, Jim Ropchan, Keunpoong Lim, Nabil E. Boutagy, Jing Wu, John C. Stendahl, Wenhua Chu, Robert Gropler, Albert J. Sinusas, Chi Liu, Yiyun Huang

**Affiliations:** 1 Department of Nuclear Medicine, West China Hospital of Sichuan University, Chengdu 610041, China; wenjie.zhang@yale.edu; 2 PET Center, Department of Radiology and Biomedical Imaging, Yale University, New Haven, CT 06520, USA; jim.ropchan@yale.edu (J.R.); keunpoong.lim@yale.edu (K.L.); j.wu@yale.edu (J.W.); chi.liu@yale.edu (C.L.); henry.huang@yale.edu (Y.H.); 3 Department of Medicine, Yale Translational Research Imaging Center, Yale School of Medicine, New Haven, CT 06520, USA; nabil.boutagy@yale.edu (N.E.B.); john.stendahl@yale.edu (J.C.S.); albert.sinusas@yale.edu (A.J.S.); 4 Department of Radiology, Washington University School of Medicine, St. Louis, MO 63110, USA; chuw@mir.wustl.edu (W.C.); groplerr@mir.wustl.edu (R.G.)

**Keywords:** PET, reactive oxygen species, in vivo imaging, automation, 2-[^18^F]fluoroethyl azide, translational study

## Abstract

Reactive oxygen species (ROS) play important roles in cell signaling and homeostasis. However, an abnormally high level of ROS is toxic, and is implicated in a number of diseases. Positron emission tomography (PET) imaging of ROS can assist in the detection of these diseases. For the purpose of clinical translation of [^18^F]6-(4-((1-(2-fluoroethyl)-1*H*-1,2,3-triazol-4-yl)methoxy)phenyl)-5-methyl-5,6-dihydrophenanthridine-3,8-diamine ([^18^F]DHMT), a promising ROS PET radiotracer, we first manually optimized the large-scale radiosynthesis conditions and then implemented them in an automated synthesis module. Our manual synthesis procedure afforded [^18^F]DHMT in 120 min with overall radiochemical yield (RCY) of 31.6% ± 9.3% (*n* = 2, decay-uncorrected) and specific activity of 426 ± 272 GBq/µmol (*n* = 2). Fully automated radiosynthesis of [^18^F]DHMT was achieved within 77 min with overall isolated RCY of 6.9% ± 2.8% (*n* = 7, decay-uncorrected) and specific activity of 155 ± 153 GBq/µmol (*n* = 7) at the end of synthesis. This study is the first demonstration of producing 2-[^18^F]fluoroethyl azide by an automated module, which can be used for a variety of PET tracers through click chemistry. It is also the first time that [^18^F]DHMT was successfully tested for PET imaging in a healthy beagle dog.

## 1. Introduction

Reactive oxygen species (ROS) are chemically reactive species, such as singlet oxygen, hydrogen peroxide, hydroxyl radicals and superoxide. ROS are involved in cell signaling which is important in the maintenance of cell homeostasis [1]. However, a sustained high level of ROS is detrimental and believed to be associated with diseases such as cancer [2,3], ischemic heart disease [4] and chemotherapy-induced cardiotoxicity [5]. Development of ROS imaging probes will enable the monitoring of ROS levels in vivo.

As a non-invasive technique, positron emission tomography (PET) imaging of ROS can assist in the detection of ROS levels in living subjects. There are several radiotracers reported for ROS imaging with PET, such as [^11^C]ascorbic acid [6], peroxy-caged-[^18^F]fluorodeoxy thymidine-1 (PC-FLT-1) [7], and the dihydroquinoline derivatives [^11^C]DHQ1 [8] and [^18^F]DHMT [9]. Among them, [^18^F]DHMT, [^11^C]DHQ1, and [^11^C]ascorbic acid have been tested in rodents. However, the short half-life (20 min) of carbon-11 limits the [^11^C]-labeled tracers from widespread clinical applications, as they require an on-site cyclotron for production. Alternately, [^18^F]DHMT is more advantageous, not only in that the longer half-life of fluorine-18 permits off-site production and transportation to multiple clinics, but also in that it can be selectively oxidized by superoxide and trapped in the cell irreversibly [9].

The usefulness of [^18^F]DHMT as an ROS imaging agent has been shown in the detection of doxorubicin-induced cardiotoxicity in a rodent model [9]. However, in the study only small-scale production of [^18^F]DHMT was reported (starting from 1.9 GBq of [^18^F]fluoride). It is challenging to scale up the production to make a large batch for multiple clinical doses, as the product is sensitive to radiolysis. Following the reported method, oxidation of the product was observed during the HPLC purification and formulation processes. In order to translate it to clinical investigations, we developed a robust and fully automated radiosynthesis of [^18^F]DHMT, which consistently yielded the product in large quantity, and with quality suitable for human use. PET imaging of the heart of a healthy beagle dog was also performed using [^18^F]DHMT. Dogs were chosen because they have been shown to be the ideal large animal for modeling human cardiotoxicity with similar chemotherapy doses [10,11,12].

## 2. Results

### 2.1. Manual Radiosynthesis under Optimized Conditions

**Scheme 1:**
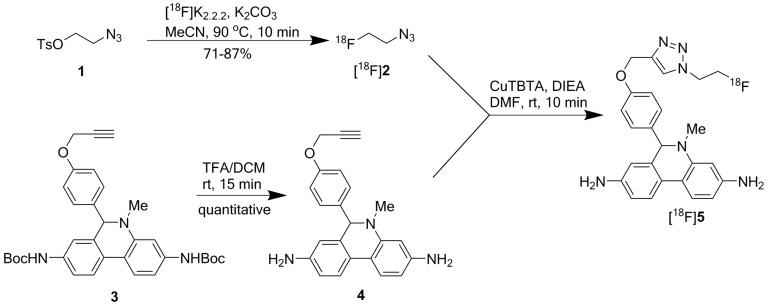
Reaction scheme for [^18^F]DHMT radiosynthesis.

[^18^F]DHMT was synthesized manually in high yield via a two-step, two-pot reaction sequence modified from the previously described method (Scheme 1) [9]. The first step was to prepare 2-[^18^F]fluoroethyl azide ([^18^F]**2**) from 2-azidoethyl tosylate (**1**). In the manual synthesis, consistently high isolated radiochemical yield (RCY) of 79% ± 8% (*n* = 3, decay corrected) was obtained for [^18^F]**2**, using 4,7,13,16,21,24-hexaoxa-1,10-diazabicyclo[8.8.8]hexacosane (Kryptofix 2.2.2 or K_2.2.2_, 5.6 mg), potassium carbonate (K_2_CO_3_, 1 mg), and anhydrous [^18^F]fluoride at 90 °C for 10 min. It was purified by passing through two stacked SepPak cartridges, eluted off with *N*,*N*-dimethylformamide (DMF, 0.5 mL), and used for the second step reaction.

**Figure 1:**
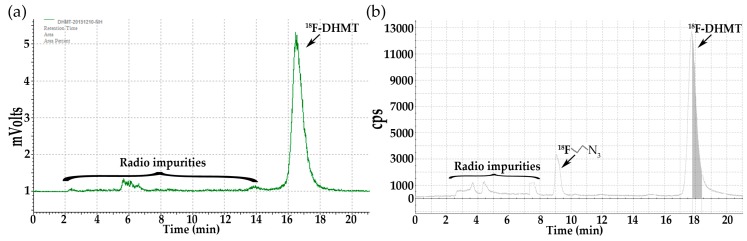
Semi-preparative radio-HPLC chromatograms for purification of [^18^F]DHMT ([^18^F]**5**). (**a**) Manual method; (**b**) Automated method.

Subsequently, Cu(I)-catalyzed click chemistry was used to construct the triazole unit of [^18^F]DHMT ([^18^F]**5**). Using a commercially available Cu(I)-stabilizing ligand, tris[(1-benzyl-1*H*-1,2,3-triazol-4-yl)methyl]amine (TBTA), the click reaction proceeded smoothly in a solvent mixture of ethanol, ammonium acetate buffer (pH = 7), and DMF (6/9/51, *v*/*v*/*v*), with 88% ± 3% (decay corrected, *n* = 2) conversion to the final product. No formation of byproducts was observed. The crude mixture was purified by semi-preparative high-performance liquid chromatography (HPLC, Figure 1a, retention time for [^18^F]**5**: 16–18 min), and formulated for intravenous injection.

### 2.2. Automated Radiosynthesis of [^18^F]DHMT

Fully automated radiosynthesis of [^18^F]DHMT with [^18^F]**2** was achieved within 77 min. The overall isolated RCY of [^18^F]DHMT was 6.9% ± 2.8% (decay-uncorrected, *n* = 7), starting from 63 to 107 GBq of [^18^F]fluoride. Specific activity was 155 ± 153 GBq/µmol (*n* = 7) at the end of synthesis.

### 2.3. Quality Control and Stability Tests of [^18^F]DHMT

Quality control results from large-scale productions of [^18^F]DHMT are shown in Table 1. The product solution was clear and colorless, with pH of about 7 (*n* = 9). The radiochemical purity of [^18^F]DHMT was 96.3% ± 1.9% (*n* = 9). The radionuclide identity was determined by measuring the radioactive decay half-life of [^18^F]DHMT, which was 110.2 ± 3.9 min (*n* = 9) and consistent with the half-life of fluorine-18. One typical production batch of [^18^F]DHMT was checked with all the quality control tests required for human use. For this production, levels of residual solvents and K_2.2.2_ were below the levels set by the US FDA and results for other tests all met the specifications for human use.

**Figure 2:**
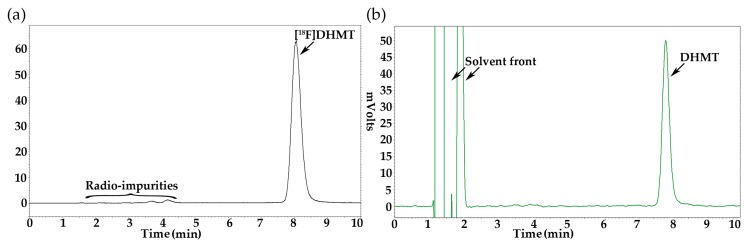
Analytical HPLC chromatograms for [^18^F]DHMT: (**a**) radioactivity detection; (**b**) UV, with co-injection of [^19^F]DHMT.

The identity of [^18^F]DHMT was confirmed by co-injection of the final radioactive product with the non-radioactive reference standard, and co-elution of the UV and radioactive peaks on the HPLC chromatogram (Figure 2). Moreover, stability test for [^18^F]DHMT produced using either the manual or automated method indicated that the radiochemical purity was maintained above 90% at 6 h after the end of synthesis.

### 2.4. Comparison of Three Methods for Radiosynthesis of [^18^F]DHMT

Table 2 shows summary of the three methods for [^18^F]DHMT radiosynthesis. Our modified manual synthesis procedure afforded [^18^F]DHMT in a total synthesis time of about 120 min, with overall RCY of 31.6% ± 9.3% (*n* = 2, decay-uncorrected, based on starting [^18^F]fluoride activity of 100–101 GBq) and specific activity of 426 ± 272 GBq/µmol (*n* = 2) at the end of synthesis. For automated radiosynthesis, [^18^F]DHMT was prepared in a total synthesis time of about 77 min. More than 4.44 GBq of the final product was obtained, with specific activity of 155 ± 153 GBq/µmol (*n* = 7) at the end of synthesis. We found a decrease in RCY (6.9% ± 2.8%, *n* = 7, decay-uncorrected) for automated synthesis compared with manual synthesis. The semi-preparative HPLC chromatogram for the purification of [^18^F]DHMT in an automated synthesis (Figure 1b) also confirmed that there was more [^18^F]**2** left unreacted when compared with manual synthesis. For manual and automated radiosynthesis, [^18^F]DHMT was produced with radiochemical purity of 93.9% ± 0.6% (*n* = 2) and 96.9% ± 1.7% (*n* = 7), respectively.

### 2.5. Animal Imaging

**Figure 3:**
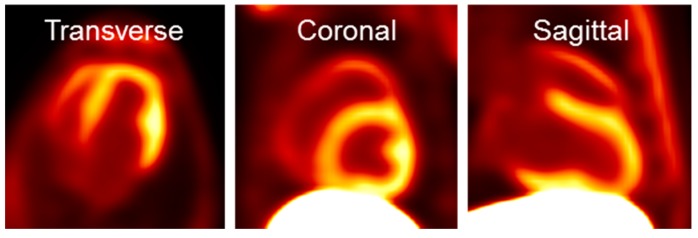
Representative [^18^F]DHMT PET images of the heart in a beagle dog.

Shown in Figure 3 are transverse, coronal and sagittal slices of PET images obtained from 60 to 90 min after intravenous injection of [^18^F]DHMT in a beagle dog. The images exhibited high myocardial-to-background ratios, especially in the left ventricle. The standardized uptake value ratio was 2.3 between the left ventricle and the blood pool, and 0.14 between the left ventricle and the liver. The right ventricle and papillary muscle were also clearly visible.

We have carried out multiple imaging studies with high specific activity [^18^F]DHMT, with injection mass of 1.05 ± 0.84 μg (*n* = 9). No adverse effects from the radiotracer were observed. Data analyses for PET imaging of healthy and diseased beagle dog models with [^18^F]DHMT are underway and the results will be published elsewhere.

## 3. Discussion

The ROS radiotracer [^18^F]DHMT was synthesized manually in high yield and with high specific activity via a two-step, two-pot reaction sequence. In our hands, the click reaction using copper(II) sulfate (CuSO_4_) and sodium ascorbate as reported in the literature failed to yield the desired product consistently. In addition, oxidation of the product during HPLC purification and post-processing was also observed. We optimized the synthesis of [^18^F]DHMT for a more consistent and reliable production, and successfully adapted it to a commercially available automated synthesis module.

The first step concerned the preparation of 2-[^18^F]fluoroethyl azide ([^18^F]**2**). We used a recently developed solid-phase extraction method instead of vacuum distillation to purify [^18^F]**2**. This solid-phase extraction method used two connected SepPak cartridges in series. [^18^F]**2** was selectively trapped on the second cartridge, and eluted out for the second step reaction. The isolated yield of [^18^F]**2** was similar to that reported (ca. 80%). This purification method is simple and reproducible, and thus particularly suitable for implementation in the automated production of ^18^F-labeled PET probes using [^18^F]**2** [13].

For the click reaction, it failed to yield the desired product consistently when we employed CuSO_4_ and sodium ascorbate as reported in the literature, probably due to the facile disproportionation of Cu(I) in aqueous solution to Cu(II) and its colloid, followed by oxidation of the product by Cu(II). In Cu-catalyzed alkyne-azide click reactions, Cu(I)-stabilizing ligands are preferred for their high efficiency during synthesis. TBTA is a commonly used and commercially available Cu(I)-stabilizing ligand. We used a cocktail of TBTA, CuSO_4_, *N*,*N*-di-isopropylethylamine (DIEA), and sodium ascorbate in the subsequent click reaction, which resulted in a highly efficient conversion and minimal formation of the oxidized byproduct.

ROS are generated by the ionizing radiation of radiolabeled products. [^18^F]DHMT is susceptible to ROS and radiolysis, which can irreversibly oxidize it to its inactive form. Therefore, it is important to reduce or eliminate radiolysis, which is challenging for large-scale production. In the previously published work, a starting radioactivity of 1.9 GBq [^18^F]fluoride was used and the involvement of radiolysis was not obvious. We found significant radiolysis of the product when we scaled up the production with large amount of starting [^18^F]fluoride (more than 63 GBq). Different HPLC purification conditions were investigated to minimize oxidation and radiolysis of the final product during the process, and to make the radiosynthesis of [^18^F]DHMT more robust and reliable for routine large-scale production. We found that addition of ascorbic acid [14,15] to the semi-preparative HPLC solvent and formulation solution prevented radiolysis during purification and formulation of the product.

We implemented the modified steps in two runs of [^18^F]DHMT using manual operations. The isolated yields of [^18^F]DHMT after semi-preparative HPLC and solid-phase extraction were slightly lower than those reported previously (40.9% and 22.3% vs. 42.8%). The radiochemical purity of the final product was 93.5% and 94.3%, respectively (Table 2).

**Figure 4:**
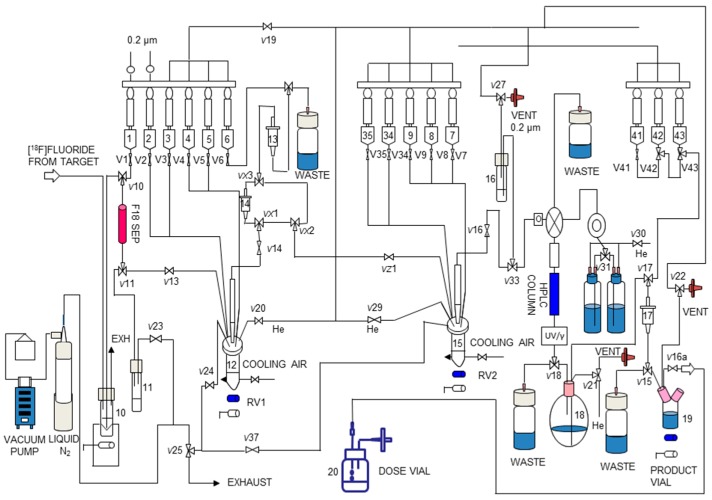
Schematic of the TRACERLab^®^ FXN Pro automated module for radiosynthesis of [^18^F]DHMT.

We then translated the two-pot, two-step synthesis of [^18^F]DHMT to a fully automated module. All steps, including the initial receiving, eluting and drying of [^18^F]fluoride from cyclotron target water, direct ^18^F-fluorination to form [^18^F]**2**, SepPak purification of [^18^F]**2**, [^18^F]DHMT formation by click reaction and, finally, preparative HPLC for product isolation, were successfully adapted to the TRACERLab^®^ FXN Pro module (GE Healthcare, Milwaukee, WI, USA). Manual radiosynthesis of [^18^F]**2** has been reported previously [16,17]. However, the isolation method for [^18^F]**2** involved distillation, which cannot be readily implemented in automated modules. Only one paper has reported a two-step, one-pot method for automated production using [^18^F]**2**. However, this two-step one-pot approach requires a large amount of alkyne precursors [18]. As mentioned above, we used a recently developed solid-phase extraction method to isolate [^18^F]**2**, which is amenable to adaptation in an automated process. To implement this method, we added a second SepPak between *VX1* and *VX3* in the module as indicated in Figure 4. To the best of our knowledge, this is the first report of implementing this solid-phase extraction method in an automated module for the purification and isolation of [^18^F]**2** for use in subsequent click chemistry. Our method can be widely applied to other automated radiosynthesis via the alkyne-azide click reaction with [^18^F]**2**.

Quality control results revealed that the [^18^F]DHMT product met all the current requirements of a radiotracer for human use (Table 1). Our methods produced [^18^F]DHMT in consistently high radiochemical purity, and with specific activity higher than that reported in the literature (Table 2). The final product from both manual and automated synthesis was stable, maintaining a radiochemical purity of more than 95% after 6 h from the end of synthesis. We found a high degree of variability in the specific activity of the final product. The water in the [^18^F]fluoride transfer line appeared to be the main factor for this variability. When the transfer line from the cyclotron to the module was cleaned and dried immediately before the radiosynthesis, a high specific activity product was obtained. When it was not cleaned and dried, a lower specific activity resulted.

A decrease in the RCY of [^18^F]DHMT was found when transitioning from manual to automated synthesis (6.9% ± 2.8%, *n* = 7, Table 2). The possible explanation for this is that a larger volume of DMF was used to elute [^18^F]**2** from the SepPak, which diluted the concentrations of the reactants in the second reactor, resulting in a less efficient click reaction. Nonetheless, the quantity of isolated [^18^F]DHMT from the automated method was sufficient for clinical applications.

Previously, [^18^F]DHMT has been validated for ROS detection through in vitro assays and in vivo imaging studies in rodents. In this study, we further applied this tracer to a large animal model. The dog images showed high myocardial-to–blood pool ratios with reasonable liver uptake. These ratios are similar to those obtained from previous studies in mice [9]. Our probe is an analog of dihydroethidium, which has been widely used for fluorescence-based detection of superoxide. Therefore, [^18^F]DHMT may have properties similar to those of dihydroethidium, such as oxidation by heme proteins. This could be the major route of probe oxidation in cardiac cells expressing high levels of heme proteins [19]. Thus, the baseline radiotracer uptake in the dog heart may be attributed to its oxidation by heme proteins. However, [^18^F]DHMT can still be used for detecting doxorubicin-induced cardiotoxicity, where ROS levels increase significantly in the heart [20].

Another point worth noting is that, as an analog of ethidium bromide, DHMT may display similar toxicity as ethidium bromide. Nonetheless, PET imaging with [^18^F]DHMT involves microdosing, with trace amount of injected mass, and toxicological effects from the radiotracer are not expected. As a matter of fact, we have carried out multiple imaging experiments in beagle dogs with injected mass dose of 1.05 ± 0.84 μg (*n* = 9) and no adverse effects were observed.

In summary, we have optimized the large-scale radiosynthesis of [^18^F]DHMT, transferred it to a commercially available automated synthesis module, and demonstrated the feasibility of using [^18^F]DHMT for PET imaging of the heart in a canine model. Future studies with this radiotracer will include its use in the detection of doxorubicin-induced cardiotoxicity.

## 4. Materials and Methods

### 4.1. General

All chemicals used were at least of analytical grade and obtained from commercial sources, unless otherwise specified. The reference standard and respective precursor for [^18^F]DHMT were synthesized following published procedures [9]. [^18^F]Fluoride was produced via the ^18^O(p, n) ^18^F nuclear reaction by irradiation of [^18^O]H_2_O (Huayi Isotopes, Toronto, ON, Canada) in a GE PETtrace cyclotron (GE Medical Systems, Uppsala, Sweden) with 16.5 MeV proton beam. Anion exchange Chromafix cartridges (PS-HCO_3_) for [^18^F]fluoride trapping were purchased from Macherey-Nagel (Dueringen, Germany). Solid-phase extraction cartridges (SepPak^®^ Light C18, SepPak^®^ Plus tC18 and Oasis^®^ Plus HLB) were purchased from Waters Associates (Milford, MA, USA).

The semi-preparative HPLC system was composed of a Shimadzu LC-20A pump (Shimadzu Corp., Kyoto, Japan) equipped with a Knauer K200 UV detector (Knauer Wissenschaftliche Geräte GmbH, Berlin, Germany), set at 254 nm, and a Bioscan radioactivity detector with Phenomenex Luna C18 (2) column (10 µm, 250 × 10 mm). Semi-preparative HPLC mobile phase was made of 35% MeCN and 65% 0.1 M ammonium formate with ascorbic acid (0.3 g/L). The flow rate was at 5 mL/min. The analytical HPLC system included a Shimadzu LC-20A pump equipped with a SPD-M20A Photodiode Array (PDA) detector (Shimadzu Corp., Kyoto, Japan) or a SPD-20A UV/Vis detector set at 234 nm, a Bioscan flow-through radioactivity detector, and a Genesis C18 column (4 µm, 250 × 4.6 mm). Analytical HPLC mobile phase was 33% MeCN and 67% 0.1 M ammonium formate with 0.5% acetic acid (pH 4.2) at a flow rate of 2 mL/min.

### 4.2. Radiochemistry

#### 4.2.1. Manual Radiolabeling of [^18^F]DHMT under Optimized Reaction Conditions

##### Deprotection of **3** to Prepare Precursor **4**

Under an argon atmosphere di-*tert*-butyl (5-methyl-6-(4-(prop-2-yn-1-yloxy)phenyl)-5,6-dihydrophenanthridine-3,8-diyl)dicarbamate (**3**, 2 mg, 3.51 μmol) in CH_2_Cl_2_ (100 μL) was added to a solution of ascorbic acid (1 mg, 5.68 μmol) in TFA (100 μL, 1.3 mmol) and deionized (DI) water (10 μL) in a 2 mL borosilicate glass V-vial wrapped with aluminum foil. After mixing, the reaction was allowed to proceed at ambient temperature for 15 min, with brief vortexing every 5 min. The solvents were dried under vacuum or argon stream at ambient temperature to afford **4** as a pale yellow solid, which was then dissolved with a solution of DIEA in DMF (200 μL, DIEA/DMF = 1/10, *v*/*v*) to form a pink solution for use in the click reaction with [^18^F]**2**.

##### Preparation of Cu(I) Catalyst

A solution of CuSO_4_-TBTA was prepared by mixing CuSO_4_ (0.4 M in 0.1 M ammonium acetate, pH = 7, 30 μL) and TBTA (0.3 M in DMF, 40 μL). The Cu(I) catalyst was prepared by mixing freshly dissolved sodium ascorbate (18 mg, 91 μmol) in water/ethanol (1:1, *v*/*v*, 120 μL) with the above CuSO_4_-TBTA solution to form a brown slurry which turned to a colorless solution upon mixing at ambient temperature.

##### Preparation of 2-[^18^F]fluoroethyl azide ([^18^F]**2**)

Preparation of [^18^F]**2** followed the literature procedures [13]. Cyclotron-produced [^18^F]fluoride in [^18^O]water was trapped on a PS-HCO_3_ anion-exchange cartridge pre-activated with ethanol (5 mL), DI water (5 mL) and dried by air. The trapped [^18^F]fluoride (100–101 GBq) was eluted slowly with 1 mL of K_2.2.2_ (5.6 mg)/potassium carbonate (1 mg) solution in MeCN/water (70/30, *v*/*v*) into a 2 mL borosilicate glass V-vial. The eluent was dried at 110 °C under argon, followed by azeotropic drying with three 1.0 mL portions of anhydrous MeCN. Compound **1** (1.5–2.0 mg) in 0.2 mL anhydrous MeCN was then added, followed by heating for 10 min at 90 °C with occasional shaking.

After cooling in ice-water (0 °C) for 1 min, the reaction mixture was diluted with DI water (10 mL) and then passed through two stacked SepPak cartridges (a SepPak^®^ Plus tC18 cartridge and an Oasis^®^ Plus HLB cartridge, both pre-activated by washing with 10 mL of EtOH followed by 10 mL of DI water). The cartridges were rinsed with DI water (10 mL × 2). The Waters Oasis^®^ Plus HLB cartridge was taken off, dried with air, inverted, then eluted with DMF (3 × 0.5 mL) to collect [^18^F]**2**. The first portion of 0.5 mL DMF was discarded, and the second portion of 0.5 mL DMF was used for the click reaction.

##### Click Reaction to Form [^18^F]DHMT ([^18^F]**5**)

The solution of [^18^F]**2** in DMF (0.5 mL) was added to a V-vial preloaded with a solution of **4** (3.51 µmol) in 200 μL of DIEA/DMF (1/10, *v*/*v*). The Cu(I) catalyst as prepared above was added to the V-vial via a syringe and the reaction was carried out at ambient temperature for 10 min with occasional shaking. The reaction mixture was then diluted with 1.2 mL of aqueous ascorbic acid solution (0.57 mM), and injected onto the semi-preparative HPLC system for purification.

The product fraction at 16–18 min was collected, diluted with 50 mL of aqueous ascorbic acid solution (0.57 mM), and loaded onto a SepPak^®^ Light C18 cartridge pre-activated by washing with 10 mL of ethanol, and 10 mL of aqueous ascorbic acid solution (0.57 mM). The SepPak cartridge was rinsed with 10 mL of aqueous ascorbic acid solution (0.57 mM). The product was then eluted off with a solution of ascorbic acid (1 mg) in 1 mL of United States Pharmacopeia (USP) grade ethanol, followed by 3 mL of USP grade sterile saline. The combined solution was then passed through a sterile 0.22 µm membrane filter (MILLEX-GV, Millipore, Millipore Corp., Billerica, MA, USA) into a vented sterile dose vial pre-charged with 7 mL of USP grade sterile saline and 200 µL of USP grade sodium bicarbonate solution (4.2%) to afford a formulated solution ready for intravenous administration.

#### 4.2.2. Automated Radiosynthesis of [^18^F]DHMT

##### Reagent Loading

Radiosynthesis of [^18^F]DHMT was performed as a two-step, two-pot reaction in a custom-modified dual-reactor TRACERLab^®^ FXN Pro synthesis module (Figure 4). Reagent loading was as follows: *Vial 1*: K_2.2.2_ (5.6 mg, 14.8 µmol) and K_2_CO_3_ (1 mg, 7.2 µmol) in 1 mL MeCN/H_2_O (7:3, *v*/*v*); *Vial 2*: anhydrous MeCN (2 mL); *Vial 3*: compound **1** (2–4 mg) in MeCN (1 mL); *Vial 4*: DI water (5 mL); *Vial 5*: DI water (18 mL); *Vial 6*: DMF (1 mL); *Vial 7*: compound **4** (2–4 mg) in 200 μL DIEA/DMF (1/10, *v*/*v*); *Vial 15*: freshly prepared Cu(I) catalyst (the same amount and preparation method as that used in the manual method); *Vial 16*: aqueous ascorbic acid solution (0.57 mM, 1.5 mL); *Vial 18*: aqueous ascorbic acid solution (0.57 mM, 15 mL); *Vial 43*: aqueous ascorbic acid solution (0.57 mM, 15 mL); *Vial 42*: ascorbic acid (1 mg) in USP grade ethanol (1 mL); *Vial 41*: USP grade saline (3 mL); *Product vial*: USP grade saline (7 mL) with USP grade aqueous sodium bicarbonate solution (4.2%, 200 µL).

##### Reaction Sequence

The optimized manual synthesis procedures for [^18^F]DHMT were transferred to an automated module (Figure 4). Synthetic protocol started with the delivery of aqueous [^18^F]fluoride to the synthesis module. [^18^F]fluoride was trapped on an ^18^F separation cartridge (*F18 SEP*) and eluted into the first reactor (*RV1*, *12*) with a solution of K_2.2.2_/K_2_CO_3_ (*Vial 1*). Azeotropic drying was performed under reduced pressure at 70 °C for 10 min and a steady stream of argon. The addition of 1 mL MeCN from *Vial 2* and subsequent drying at 100 °C for 5 min was repeated twice. After drying, the solution of compound **1** (*Vial 3*) was added into *RV1* and heated at 85 °C for 15 min to form [^18^F]**2**. After cooling down to 35 °C, 18 mL of DI water (*Vial 5*) was added and the content in *RV1* was passed through the two SepPak cartridges (*14* and *13*) sequentially, followed by washing with 5 mL of DI water (*Vial 4*). The intermediate was eluted off SepPak *13* with 1 mL of DMF (*Vial 6*) into the second reactor (*RV2*, *15*). The precursor **4** in 0.2 mL of DIEA/DMF (*Vial 7*) was added and the reaction was carried out at ambient temperature for 10 min. The crude reaction solution was transferred to *Vial 16* and loaded onto the semi-preparative HPLC system for purification.

The desired product fraction was collected in a round bottom flask (*Vial 18*), diluted with 15 mL of aqueous ascorbic acid solution (0.57 mM), and passed through a SepPak^®^ Light C18 cartridge (*17*). The cartridge was washed with 10 mL of aqueous ascorbic acid solution (0.57 mM) from *Vial 43*. The product was eluted off the SepPak with a solution of ascorbic acid (1 mg) in USP grade absolute ethanol (1 mL) (*Vial 42*), followed by 3 mL of USP grade saline (*Vial 41*), into the *Product Vial 19*, which was preloaded with 7 mL of USP grade saline and 200 µL of sodium bicarbonate aqueous solution (4.2%). Terminal sterilization was performed by passing the final formulated solution in the *Product Vial* through a 0.22 µm sterile membrane filter into a sterile *Dose Vial* (*Vial 20*).

### 4.3. Quality Control and Stability Test

Visual inspection of [^18^F]DHMT ([^18^F]**5**) was done behind a shielded L-block. The filter membrane integrity test was done by a bubble pressure test. The filter was first rinsed by 10 mL of DI water and then the inlet of the filter was attached to argon gas and the outlet of the filter was immersed in water. The argon flow was slowly increased until there was continuous bubbling and the pressure reading recorded as bubble pressure. Solution pH was measured by spotting the product solution onto a strip of pH paper. Residual organic solvent analysis was conducted by gas chromatography (GC) analysis on a Restek Rtx-200 capillary column (60 m × 0.53 mm). Endotoxin level was tested using the LAL test on the Endosafe-PTS device (Charles River Laboratories, Charleston, SC, USA). After decay of radioactivity, post-release sterility tests were performed by incubation of the final product solution with culture media for 14 days. The residual K_2.2.2_ test was done by visually comparing the spotted sample of product solution with a standard solution of K_2.2.2_ on the same TLC plate after development and iodine staining. Radionuclide identity was confirmed by measuring the half-life of [^18^F]**5**. Chemical purity, radiochemical purity, and specific activity of [^18^F]**5** were determined by HPLC analysis of the final product solution using the conditions described above. The identity of the labeled compound [^18^F]**5** was confirmed by co-injection of the product solution with the reference standard **5**. Stability tests for [^18^F]**5** from both manual and automated radiosynthesis methods were performed at 2, 4, and 6 h after the end of synthesis by checking the radiochemical purity using the same analytical HPLC conditions.

### 4.4. Animal Preparation and Surgery

A healthy beagle dog (11 kg) was sedated with propofol (7.5 mg/kg) via intravenous injection in the foreleg cephalic vein, and then intubated for mechanical ventilation (Veturi; Cardiopulmonary Corp., Milford, CT, USA) and anesthesia maintenance. Anesthesia was maintained with 1%–3% isoflurane, 65% nitrous oxide, and 35% oxygen. The level of anesthesia was determined by monitoring heart rate, blink reflex and jaw tone. Blood gases, electrolytes (VetStat Electrolyte and Blood Gas Analyzer, IDEXX Laboratories Inc., Westbrook, ME, USA) and hematocrit were serially measured throughout the study and ventilator settings were adjusted accordingly to maintain physiological conditions. Cardiac rhythm and rate (ECG), oxygen saturation (pulse oximeter), and body temperature (rectal temperature probe) were monitored via a Phillips InteliVue MP50 monitor. A small femoral cut-down (4 cm) was performed and a 6F introducer sheath was placed in the right femoral artery to monitor pressure.

### 4.5. Animal PET/CT Imaging

The dog was placed feet first in a lateral decubitus position for imaging on a Siemens mCT PET/CT scanner. ECG leads and a respiratory gating belt were placed for cardiac and respiratory motion corrections, respectively. Prior to the PET scan, a non-contrast CT scan (120 kV, 11 mAs, 2.0 mm slices) was performed during temporary detachment from the ventilation to limit respiratory motion artifact. Following the CT scan, 0.185 GBq of [^18^F]DHMT was injected via the femoral vein and a 2 h dynamic PET scan was performed.

All animal experiment protocols were approved by the Institutional Animal Care and Use Committees at the Yale University School of Medicine (protocol: 2014-11623), according to the guiding principles of the American Physiological Society on research animal use.

## 5. Conclusions

We reported an optimized and automated radiosynthesis of [^18^F]DHMT for large-scale production. This study is the first demonstration to produce 2-[^18^F]fluoroethyl azide with an automated module, which can be used to produce a variety of other PET tracers through click chemistry. The production process for [^18^F]DHMT yielded the product in reliably high radiochemical purity and quality suitable for human use. PET studies demonstrated the feasibility of using [^18^F]DHMT for imaging of the heart in a canine model.

## Figures and Tables

**Table 1:** Quality control results of [^18^F]DHMT.

Test	Release Criteria for Human Use	Result
Visual inspection (*n* = 9)	Clear, colorless	Clear, colorless
Solution pH (*n* = 9)	4.5–8.5	7
Residual solvent test (*n* = 1)	MeCN < 0.41 mg/mL, DMF < 0.88 mg/mL	Pass
K_2.2.2_ test (*n* = 1)	<0.05 mg/mL	Pass
Endotoxin test (*n* = 1)	<17.5 EU/mL	<5 EU/mL
Sterile filter integrity test (*n* = 1)	Bubble point pressure > 50 psi	Pass
Sterility test (*n* = 1)	No bacterial growth	Pass
Radiochemical purity (*n* = 9)	>90%	96.3% ± 1.9%
Radionuclide identity (*n* = 9)	Measured *t*_1/2_ between 98.8 to 128.8 min	110.2 ± 3.5 min

**Table 2:** Comparison of three methods for radiosynthesis of [^18^F]DHMT.

Method	RCY * (%)	Radiochemical Purity (%)	Specific Activity (GBq/µmol)	Synthesis Time (min)
Literature [9]	42.8	90–100	13–24	120
Manual (*n* = 2)	31.6 ± 9.3	93.9 ± 0.6	426 ± 272	~120
Automated (*n* = 7)	6.9 ± 2.8	96.9 ± 1.7	155 ± 153	~77

* Decay-uncorrected RCY calculated based on the isolated product and starting [^18^F]fluoride.
